# Genetic counseling in the context of Bangladesh: current scenario, challenges, and a framework for genetic service implementation

**DOI:** 10.1186/s13023-021-01804-6

**Published:** 2021-04-09

**Authors:** Mohammad Jakir Hosen, Saeed Anwar, Jarin Taslem Mourosi, Sourav Chakraborty, Md. Faruque Miah, Olivier M. Vanakker

**Affiliations:** 1 Department of Genetic Engineering and Biotechnology, School of Life Sciences, Shahjalal University of Science and Technology, Sylhet, 3114 Bangladesh; 2 Department of Medical Genetics, Faculty of Medicine and Dentistry, University of Alberta, 8440 112 St. NW, Edmonton, AB T6G 2R7 Canada; 3 Department of Biology, The Catholic University of America, 620 Michigan Avenue NE, Washington, DC 20064 USA; 4 Research and Development Laboratory, Globe Biotech Limited, Tejgaon, Dhaka, 1208 Bangladesh; 5 Center for Medical Genetics, Ghent University Hospital, Corneel Heymanslaan 10, 9000 Ghent, Belgium

**Keywords:** Genetic service, Disease burden in Bangladesh, Neonatal screening, Genetic counseling, Legal and social issues, Collaborative research

## Abstract

With the advancements in genetics and genomics in the twenty-first century, genetic services have become an integral part of medical practices in high-income and upper-middle-income countries. However, people living in low and lower-middle-income countries (LICs and LIMCs), including Bangladesh, are rather underprivileged in receiving genetic services. Consequently, genetic disorders are emerging as a significant public health concern in these countries. Lack of expertise, high expense, the dearth of epidemiological data, insufficiently updated medical education system, poor infrastructure, and the absence of comprehensive health policies are the main factors causing people living in these countries not having access to genetic services. In this article, the authors took benefit from their professional experience of practicing medical genetics in the area and reviewed existing literature to provide their opinions. Particularly, it reviews the current knowledge of genetic disorders' burden and their causative factors in Bangladesh. It focuses on why providing genetic services is challenging in the context of the country's cultural and religious sentiment. Finally, it proposes a physician-academician collaborative framework within the existing facility that aims to tackle the challenges. Such a framework could also be useful for other LICs and LMICs to address the challenges associated with providing genetic services.

## Introduction

To date, more than 6500 inherited disorders have been identified globally, and common genetic diseases are increasingly emerging as a major health challenge [1, 2]. Early and accurate diagnosis of these diseases is critical to optimal care for patients [3–5]. However, the diagnosis of genetic diseases can be challenging and contingent upon the understanding of that particular disease's molecular etiology [5]. Although our understanding of many human diseases has advanced significantly in the post-genomic era, knowledge of most genetic diseases is still limited. This is primarily because most of these diseases are rare, clinically heterogeneous, and the reality that there are thousands of genetic diseases with only a few patients been identified for which causative factors still have to be deciphered [6–8]. Besides, the expertise, infrastructure, and expenses required for genetic disease management are not frequently available. Additionally, several complex and interrelated strategic, socio-cultural, and ethical issues, including the lack of a non-directive delivery approach for genetic services, inadequate psychosocial support, familial and religious values, and socio-cultural stigma, often influence the uptake and understanding of genetic services [9].

Currently, available management strategies for most genetic and multifactorial diseases are rather palliative, as curative treatment strategies are not commonly available [10]. Alongside, genetic diseases require continuous medical follow-up and therapy, which is highly expensive and, especially for gene therapy—experimental in most cases [11, 12]. To face the challenges with genetic diseases, upper-middle-income and high-income countries have made significant progress in human and medical genetics. These countries have started many government and non-government collaborative and consortium-based initiatives to understand, manage, and cure these diseases better [6, 13, 14]. In contrast, the low and lower-middle-income countries (LICs and LMICs), representing a large proportion of the human population, have either taken very scarce initiatives or no initiatives yet. This, in turn, has added to the existing disparity in the low-income and lower-middle-income countries (LICs and LMICs), including Bangladesh, as access to health care is limited, and progress in genetic services is negligible compared to upper-middle-income and high-income countries (UMICs and HICs) [15–17].

In this review, we provide an overview of the challenges associated with the provision of genetic services in the LICs and LIMCs, specifically in Bangladesh. We have discussed the current health-care system in Bangladesh and reviewed the incidence of genetic and multifactorial diseases in the population, cultural factors impacting genetic disease prevalence, and the role of religious practices in setting boundaries on acceptable management guidelines for pregnancies identified to be affected with a genetic disease. We endeavored the key challenges to harnessing genetic information in order to reduce the burden of genetic diseases in Bangladesh. This article proposes a framework for delivering genetic services in the country, which includes dissemination of genetic literacy to the population at large and fostering international collaboration between local physicians, academics, and international experts. The proposed framework could be a model for implementing genetic services in other LICs and LMICs having similar health and cultural status.

## Socio-demographic features and health system of Bangladesh

Health-related socio-demographic features of Bangladesh are listed in Table 1. Bangladesh has a population of around 160 million (~ 2.2% world’s total population), representing the world’s most densely populated country. With 90% of the population being Muslim, Islam is the largest religion. The current life expectancy of the Bangladeshi population is approximately 70 years compared to 75 years in developed countries [18]. The infant death rate is high; approximately 240 deaths per 100,000 live births. Only 25% of the mothers go to a health care facility to give births, putting them and their babies at risk. Malnutrition is a critical health issue in Bangladesh, with a high incidence of anemia, growth, and weight deficiencies [19]. The “Global Burden of Disease” study recently analyzed 33 health-related sustainable development goal indicators for 188 countries, where Bangladesh was positioned at 151st place [20]. The overall health system in Bangladesh mainly relies on the government and public sector, as less than 3% of the GDP is allocated for health services [21]. Unfortunately, until now, there is no health insurance policy in Bangladesh.

**Table 1:** Health-related socio-demographic features of Bangladesh

Broad area	Indicators	Value
Population	Total	162.6 million [156]
	Density (population/km^2^)	1015 [157]
	Average death per 1000 people	5.4 [158]
Life expectancy	Male	66.64 years [157]
	Female	68.79 years [157]
No. of doctors	Per per 10,000 people	3.9 [156]
No. of hospital beds	Per 10,000 people	4 [21]
No. of physicians	Total	11,300 [159]
Nurses	Per 10,000 people	1.07 [18]
Health services	GDP spent on healthcare	2.8% [18]
	Health expenditure as a % government budget	7.4 [18]
	Out-of-pocket expenditure for health	65.9% [18]
	Percent coming from development aid/partners	8 [18]
	Per capita total expenditure on health	23 [18]

*GDP* gross domestic product, *no* number

There is no recognized Government medical genetics center available in Bangladesh, except for one small national Department for Forensic Medicine in Dhaka (Criminal Investigation Department, Police Head Quarter, Dhaka). Further, the current medical education system (MBBS degree, basic degree of a medical student) fails to form geneticists in Bangladesh; essence to incorporate knowledge of genetics in both undergraduate and postgraduate levels of medical curriculum [22]. As the number of doctors compared to the whole population is insufficient, one can already start his career as a medical doctor after 5 years of general medical education (MBBS). In Bangladesh, no general practitioners keep an overview of and manage a patient's medical history. Moreover, there is neither a proper document storage system nor an information exchange system from hospital to hospital or from physician to physician.

## Epidemiology of genetic diseases in Bangladesh

As in many LIC and LIMCs, Bangladesh has no comprehensive epidemiological data on the prevalence of most genetic diseases. Though inhabitants suffer from many hereditary disorders and congenital malformations [23], it is difficult to collect precise prevalence data due to poor registration, significant diversity of conditions and also because many disorders remain undiagnosed [24].

Despite the fact that Bangladesh lies in the so-called thalassemia belt (roughly 3.6 million carriers of thalassemia) and hereditary hemoglobinopathies are the most common genetic diseases in Bangladesh, the information on different aspects, including epidemiology, clinical course, mortality, complications, and treatment outcomes are scarce due to the lack of definitive national data [15, 25, 26]. Considering the estimated global prevalence of many genetic diseases, it can be assumed that the variety of common and rare genetic diseases in Bangladesh is considerably larger.

### Common diseases with a genetic predisposition

Over the last decades, mortality caused by the ‘fatal four,’ i.e., cardiovascular disease (CVD), cancer, chronic respiratory diseases, and diabetes mellitus (DM), is increasing at an alarming rate. In all four, genetic risk factors are being identified [27, 28].

CVD has been reported as the leading cause of mortality in Bangladesh, which probably has the highest CVD incidence in the South of Asia [27, 29, 30]. Only a limited number of small-scale studies are available regarding the epidemiology of CVD in Bangladesh. The age-standardized CVD mortality rates have increased by at least 30 fold among males and 47 fold among females from 1986 to 2006 in rural Bangladesh [31]. Bangladeshi immigrants living abroad have been reported to suffer more severe and more fatal cardio- and cerebrovascular disease compared to Caucasians (e.g., in the UK, Bangladeshi men had 112% higher coronary artery disease mortality and 220% higher stroke mortality) [32, 33]. Despite that individual CVD risk factors have not been fully studied, the increased susceptibility of South Asian ethnicities to CVD is due to three mechanisms: disease-related mutations, increased prevalence of susceptibility alleles, and different gene-environment interactions [34, 35].

Cancer is a major health burden worldwide and the sixth common cause of mortality in Bangladesh [36]. By 2030, over 9 million cancer patients are assumed to die in the LICs and LIMCs [37]. There are 1.3–1.5 million cancer patients in Bangladesh, with about 0.2 million newly diagnosed patients each year [34], with lung cancer in males and cervical and breast cancer in females being most frequent (38% of all cancer cases) [36]. Somatic molecular genetic testing is available mainly for the evaluation of the Philadelphia chromosome in the context of hematological malignancies, thanks to the help of a foreign diagnostic center [38]. Genetic testing for germline pathogenic variants to identify common familial cancer syndromes such as hereditary breast and ovarian cancer, or Lynch syndrome [39], is not available at all [40].

DM is considered a major public health issue in Bangladesh due to its high prevalence, which may exceed 16.8 million by 2030 [41]. A study in 2014 confirmed that more than half of the people with diabetes were not diagnosed, and only about 40% of the patients received regular treatment [42]. The high incidence of DM is associated with disability and mortality due to stroke and cardiovascular disease, renal insufficiency, neuropathies, and visual impairment [42, 43]. In Bangladesh, older age, female gender, obesity, and a positive family history of DM were significantly associated with insulin resistance (type 2 DM) [44].

### Other genetic diseases

In 2013, 12% of mortality in neonates and children of under-five years of age was estimated to be due to congenital anomalies [45]. As in other countries, the most common birth defects are congenital heart malformations, associated with significant mortality in Bangladesh [46]. A prospective study held at the Combined Military Hospital revealed that 2.5% of all live births had congenital heart diseases (CHDs) [23]. Among different CHDs, the atrial septal defect (26%), ventricular septal defect (16.9%), patent ductus arteriosus (18%), tetralogy of Fallot (14%), and pulmonary stenosis (7.75%) were most common [23]. Working at the Dhaka Shishu Hospital, Hussain et al. also reported a very similar result [46].

The entire South Asian region, including Bangladesh, is considered a hotspot of hemoglobinopathies [47]. As mentioned earlier, inherited ß-thalassemias, as well as sickle cell anemia and hemoglobin E (HbE), are the most frequent single-gene disorders in Bangladesh. It is estimated that approximately 2,500 babies with thalassemia major are born in Bangladesh each year [26]. Several population studies, of both small and large scale dimensions, have suggested that Bangladesh is home to most of the South Asian variants of ß globin with other novel and rare variants in this region, which includes the Mediterranean and Far-Eastern type mutations [48–50]. Also, most of the common variants for α-globin that leads to α-thalassemia are present in Bangladesh [51]. The presence of the novel and rare variants indicates the heterogeneity and endemical nature of thalassemias in the country.

Down syndrome is the most frequently reported chromosomal disorder in Bangladesh [15, 52]. Other reports include Patau syndrome [53], Turner syndrome [54, 55], intersex disorders (absence of typical binary notions of male or female bodies, e.g., due to aneuploidy) [56, 57], and Klinefelter syndrome [55, 58, 59]. Among rare conditions, previous research reported several cases of Marfan syndrome [60], scleroderma [61, 62], multiple epiphyseal dysplasia [63], Cutis laxa [64], and Bart’s syndrome [65].

## Predisposing factors for genetic diseases in Bangladesh

Consanguineous marriage is considered one of the predisposing factors behind the burden of genetic diseases in Bangladesh [66]. It significantly increases the prevalence of autosomal recessive genetic conditions in Bangladesh [67, 68]. Consanguinity increases the prevalence of rare genetic and congenital anomalies and nearly doubles the risk for infant mortality [69–71]. It is well known that a higher risk of recessive genetic disorders is present in children of consanguineous parents. Rates of 2–4% are widely quoted for autosomal recessive disorders in the children of first cousins; at an individual family level, the observed risk can be up to 25% or higher [72]. In addition, many studies have so far suggested a positive correlation between the rate of congenital heart disease with the occurrence of consanguineous marriage [71, 73].

In general, consanguinity is influenced by geographic, demographic, religious, cultural, and socio-economic factors. This custom is common in the Muslim community throughout the world. Islam allows but always discourages first cousin marriages [74]. In Bangladesh, very few studies have attempted to determine the current prevalence of consanguinity or its effects on rare genetic disorders. We recently reported that the prevalence of consanguineous marriage in Bangladesh is 6.64% [66]. However, a previous study reported that the prevalence of consanguinity in the extreme southeast part of the country is 17.6%, compared to 6.7% in the Matlab area of Chandpur district [75, 76]. It was reported that consanguineous marriage has a strong influence on reproductive behavior and is associated with several monogenic and multifactorial diseases and congenital anomalies, e.g., including bronchial asthma, hearing defect, heart diseases, and sickle cell anemia, in Bangladesh [66]. Besides, coronary lesions and hypertension are more prevalent when consanguinity is present [77, 78].

In addition to genetic factors, environmental factors play a crucial role in the occurrence of genetic diseases. Environmental factors can lead to disease-causing or putative de novo mutations [79–82]. In Bangladesh, pollution of the air, ground-water resources, and rivers with cadmium, lead, arsenic, and other chemical substances may play a role in the increased incidence of some particular health problems [83, 84].

## Current scenario of genetic testing and counseling in the context of Bangladesh

Genetic counseling is considered an essential part of the management of patients and families in which a genetic disorder has been diagnosed or who are at risk for such a disease. It provides patients the necessary information about the disease and their genetic profile. Also, it calculates the risk or probability of a genetic disorder appearing in their family, and provides support, diagnosis, and management facilities and—if available—treatment. In LMICs, such as Bangladesh, most inhabitants are unaware of the inheritance patterns and risk factors of genetic diseases, which poses additional challenges for implementing genetic services for them. Thus, along with developing infrastructure for genetic service delivery, comprehensive public awareness programs should be introduced throughout the country to spread knowledge on the deleterious effects and possible preventive measures of genetic diseases, and the role of consanguinity. Moreover, suitable measures need to be taken to ensure proper treatment and follow-up for patients with genetic diseases.

### Premarital counseling

Premarital counseling is regarded as the first step to minimizing genetic disease transmission from parents to offspring [85]. It is imperative to reduce the transmission of autosomal recessive disorders in the context of consanguineous marriages, the genetic burden of which Bangladeshis do not usually comprehend properly [66, 85]. Awareness campaigns are likely to contribute a major part to the success of premarital counseling in such circumstances [86]. Such counseling has already become popular in the Middle East, aiming to identify ß-thalassemia carriers among couples planning to marry [87]. Premarital screening could reduce the ß-thalassemia burden in these regions, preventing up to 95% of affected births [88]. Similar programs were implemented in Italy, Iran, and Greece, achieving a significant reduction in ß-thalassemia births [89, 90]. Thus, premarital counseling can be regarded as one of the most effective measures to reduce the incidence of genetic disease associated with a high carrier frequency in the LICs and LMICs, like Bangladesh.

### Prenatal screening

Prenatal screening could be followed by premarital screening as the second step for early diagnosis of any congenital abnormalities [91]. The chromosomal aneuploidies account for around 95% of live-born chromosomal abnormalities, often tricky due to timing, expense, sensitivity, and ethical issues [92]. To date, no prenatal screening system is available in Bangladesh. An important reason for the lack of prenatal screening is the technical inability of most Bangladeshi Hospitals to perform the confirmatory molecular/genetic tests for chromosomal abnormalities, e.g., Down syndrome. In many cases, diagnosis is mainly depending on the general clinical features. It should be taken into account that while most pregnancies in city areas are often followed carefully, perhaps by using modern sonography techniques, this is only the rare case in rural areas.

### Newborn screening

Newborn screening (NBS), finally, is a simple test at birth that can identify genetic conditions that may affect a child's long-term health or survival but are treatable at birth [93]. In many countries, NBS programs have dramatically improved the morbidity and mortality associated with the disorders screened [94, 95]. However, in many other countries, mostly LICs and LMICS, NBS programs were not as successful- not because of the diagnostic accuracy but for sociocultural and health-education related reasons. Indeed, successful implementation of NBS requires a comprehensive system of education, follow-up, diagnosis, management, and evaluation that must be institutionalized and sustained within public health systems. In most cases, NBS programs' implementation is challenging in the LICs and LMICs [96]. In the last two decades, some LICs and LIMCs in the Asia Pacific, Middle East, and North African regions initiated NBS; however, the progress is slow due to various factors, including poor economies, weak public health policies, and mediocre delivery methods [16, 97]. Especially in the conservative cultural setup of the Indian subcontinent, like in Bangladesh, many people find clinical procedures as taboo-like phenomena and do not prefer to visit hospitals for labor purposes [98]. Even those who decide to visit the hospital, rarely allow sampling by any invasive techniques, e.g., blood collected by heel pricking of the newborn. As a result, the collection of samples from newborns is challenging [99]. In some specific regions of Bangladesh, the government tried to run a pilot project for NBS, especially for congenital hypothyroidism, but no nation-wide institutionalized and sustainable NBS program is currently available [100].

## Genetic testing facilities in Bangladesh

To identify the biochemical, chromosomal, and/or molecular etiology of a genetic disease, only a few private hospitals, clinics, and diagnostic centers are recently trying to provide comprehensive lab testing in Bangladesh. Except for some minimal facilities that can perform such diagnostic tests, their diagnostic service is mainly confined to collecting and preparing patient samples and exporting them to foreign diagnostic centers, mostly in India, where the tests are performed. There are currently neither molecular labs nor community projects run by the government of Bangladesh nor medical genetics departments. The limited molecular genetic testing that happens in the country is predominantly restricted to sophisticated laboratories like in the public universities, the International Centre for Diarrheal Disease Research (ICDDRB), the Bangladesh Institute of Research and Rehabilitation in Diabetes, Endocrine and Metabolic Disorders (BIRDEM) [39]. These institutions do have laboratory set-ups for modern molecular genetic techniques to make reliable clinical diagnoses of most of the common genetic conditions. The studies these institutions carry out are, notwithstanding, not used as diagnostic or prognostic tools; they instead serve the academic interest. Most of the molecular genetic testing facilities are yet to be widely available for clinical and diagnostic purposes. Recently, a few private hospitals and diagnostic centers have taken the initiative to establish a self-sufficient molecular diagnostics laboratory in Bangladesh, but so far, they mainly collect samples with molecular analysis being performed abroad if possible. Besides, some twenty universities in Bangladesh now have departments of Genetic Engineering and Biotechnology, and Biochemistry and Molecular Biology, or other related departments with molecular genetics laboratory facilities. Graduates of these departments receive a decent extent of training on genetics, molecular techniques, and diagnostics; however, they often are neglected to contribute to the diagnostic centers (as molecular biologists, pathologists, and laboratory geneticists), and broadly, the health sector [101]. Also, if collaborations could be achieved with physicians who can identify the possible genetic risk of a patient, a significant amount of molecular diagnostic works can be done in these university facilities.

## Current education of medical professionals

The curriculum of medical colleges in Bangladesh is not sufficiently focused on the study of genetics and heritable disorders as well as modern diagnostic tools and strategies. The undergraduate medical curriculum of Bangladesh, introduced in 2012, includes only 4-h of basic genetics in Anatomy class [102]. The post-graduate curriculum also includes only basic genetics, insufficient to deliver proper knowledge on molecular genetic research and advanced molecular diagnostics [22]. At Bangabandhu Sheikh Mujib Medical University, the only medical university in Bangladesh, there is a small facility for genetic disease research. As the physicians in Bangladesh do not get sufficient exposure to medical genetics academically, it is often difficult for them to provide accurate and straightforward information to patients about genetic conditions and help them make decisions. Also, no diagnostic and pathology staff and the paramedics (including medical technologists, nurses, and care workers) receive any training focusing on genetic diseases. As a result, the medical professionals' lack of expertise to deal with genetic conditions is another major challenge in genetic counseling provision in Bangladesh.

## Ethical, legal, and social issues in genetic counseling

Rapid evolution in technological possibilities to analyze the human genome and increasing knowledge of its function provides a wealth of new options for dealing with genetic diseases, but also several issues that must be addressed by the genetic services provider, patients, as well as the society. Artificial insemination, genetic screening on embryos, in vitro fertilization, sex selection during prenatal testing, surrogate motherhood, fetal transplantation, and gene therapy raises ethical issues; genetic counselors can play a vital role in the way they are understood and acted upon.

### Confidentiality and privacy

A few social and ethical issues accompany the practice of genetic counseling, related to the confidentiality and privacy protection of the patients. The need for confidentiality of sensitive information about a person’s family history, carrier status, and particularly, the risk for a genetic disease, which can lead to social stigma, calls for disciplinary control over medical practitioners, as is done by the Medical and Dental Council of Bangladesh (BMDC). Although this organization is in place, attempting to control malpractice, misbehavior, and violation of the ethical code by medical practitioners, people opt to report directly to court instead. BMDC is a semi-autonomous body under the ministry of health and family welfare; one of its departments is the ethical committee whose function is to review ethical aspects of research protocols before approval. But the BMDC mostly deals with research of medical colleges situated in Dhaka. Further, there are issues regarding its own poor functioning and lack of monitoring [103, 104].

### Religious issues

Among factors that affect a couple’s willingness to receive genetic counseling, religions issues that guide marriage habits and reproductive behavior are important. Some aspects of genetic counseling, such as the discussion of a diagnosis, prognosis, and preconceptual options, are quite often sensitive in a like Bangladesh where educational status is suboptimal, and the majority of inhabitants is Muslim, guided by Islam [105, 106]. Therefore, the change of social context requires careful consideration, as exemplified by the use of prenatal screening, which has a few limitations since therapeutic abortion is only permitted within 120 days from conception and only under special circumstances [87, 107]; elective abortion is generally unacceptable [108, 109].

The development of the preimplantation and prenatal diagnosis of genetic disorders has offered couples an alternative to prenatal diagnosis to avoid selective abortion. According to Islamic jurisprudence, if diagnostic tests prove that a fetus is affected by an untreatable life-threatening disease or carries some severe disability, therapeutic abortion is permissible and lawful, given that the pregnancy is terminated before the time of breathing the soul, i.e., within 120 days of gestation [110]. Muslim jurists agree that genetic disorders' preimplantation diagnosis is permissible on medical grounds only if it intends to benefit the couple and perhaps also the fetus, do not conflict with other Islamic rulings, uses materials not derived from the forbidden sources, and does not aim just to screen embryos with undesired traits [110–112]. Genetic manipulation for cosmetic reasons affecting future progeny is strictly prohibited [113]; hence eugenics and dysgenics are impermissible while allowing gene therapy for therapeutic purposes [114, 115]. Sex selection of babies is Islamically permissible only when certain medical conditions—which cause physical and psychological burden—are associated with specific sex [113]. The religion teaches serenity and acceptance of God's will and does not prefer transgenic babies, even though it has been considered in some 'fatwas.' As such, the religious viewpoints on the matter remain vague and open to interpretation.

The recent progress of genome editing technologies, e.g., CRISPR/Cas9 and antisense technology, has set off new horizons for translational research and treatment of genetic and multifactorial diseases, including cancer, AIDS, thalassemia, and muscular dystrophy [116, 117]. Germline gene editing may result in heritable changes in the human genome; hence the argument of whether it should be permitted necessitates intense and comprehensive discussions. In the Islamic perspective, human germline gene editing would be considered lawful only if it (i) is used solely for medical purposes, (ii) does not bring any impairment to the parents, the offspring, and the society, and (iii) has strict regulation established to ensure abuse of the technology in human genetic enhancement [114].

Good parental information is essential for the success and ethical acceptability of any newborn screening program. The choice that a couple makes about their progeny heavily depends on the stage at which information about the risk of genetic disorder becomes available to them, and thus on the timing of the prenatal diagnosis, which has come to be known to be barely sufficient, in our experience.

### Legal issues

In many low and lower-middle-income countries, the options discussed during genetic counseling to prevent inherited genetic disorders, such as prenatal diagnosis, carrier screening, and selective abortion, often have legal prohibitions [113]. For instance, all Arab countries, except for Tunisia, have banned selective termination of pregnancy [118]. Only recently, most Islamic countries exhibit leniency towards abortion for genetic reasons, especially when it can be done fairly early in pregnancy. A classic example is Iran’s systematic approach to community consultation, wherein it provides premarital screening for thalassemia, recording the reactions of carrier couples, and responding with a fatwa permitting termination of pregnancy for serious congenital disorders before 16 weeks of pregnancy. Similar open-mindedness is adopted by countries such as Pakistan [119]. In Bangladesh, there is no legal framework regarding genetic testing and preventive measures. The country still is governed by the penal code 1860, where induced abortion is strictly illegal unless the woman’s life is in danger [120]. There are no indications for career screening and preventive measures in response to genetic diagnosis. Before the initiation of any genetic services in the country, contemporary strategic plans are to be devised to address these ethical and legal issues.

### Health insurance facilities in Bangladesh

As mentioned earlier, the uptake of genetic services is used to be constrained because of the expenses of genetic testing. Coverage of the cost of genetic testing by health insurance is beneficial in ensuring genetic services not being hampered due to cost issues [121]. However, even in the UMCs and UIMCs, insurances often do not cover everything relevant to genetic services [122–124]. The relation between genetic services and insurance is often debated on the ground that genetic information of an individual is sensitive, personal, confidential and it may alter one's perception of future medical risk and insurance agencies might need to access this information, which would hurt the individuals privacy [125, 126]. Most American and European countries, e.g., Canada, Finland, and France, have clarified their position regarding genetic services and insurance [126]. These countries, by now, have distinct sets of rules and regulations on insurance coverage to ensure proper genetic services to their citizens. In the Islamist countries, very few have a set of defined regulations, including for insurance coverage [127]. To note, Islam, with some special regulation been applied, allows insurance policies for medical and genetic causes [127, 128].

Over the years, Bangladesh has built a good foundation for social security; however, unplanned growth of the social safety net portfolio has caused fragmented implementation, with 123 programs under 25 ministries [129]. Besides, approximately 44 farms are listed as insurance companies, mainly focusing on general, life, and accidental insurance [130]. However, health insurance is not a widely used facility in Bangladesh, and the contribution of social security and private health insurance in healthcare payments is insignificant and negligible [131]. All civil servants are enrolled in several limited Government insurance schemes. A sum of BDT 700 (~ 8.4 USD) is paid to each Government employee monthly as a medical allowance. About BDT 90 (~ 1.1 USD) is taken from the salary for group insurance, for which a maximum of BDT 100.000 (~ 1200 USD) can be claimed for expensive medical treatments once in a lifetime [132].

A few private companies have begun to provide health insurance for their employees [140], which may cover hospital-room rent, consultation fees, routine investigations, medication, surgery, ancillary services, and cash benefits for hospitalization in public hospitals, with special payments applicable for normal delivery and caesarian delivery. However, due to limited knowledge of and awareness of genetic diseases in Bangladesh, Bengali health insurance does not cover the medical expenses for genetic services. It is hoped that more comprehensive health insurance policies that include the care for patients and families with a genetic disorder will arise with the necessary improvements in the diagnostic facilities and the economic status of the population of Bangladesh.

## Perspective framework towards implementation of genetic services

The integration of genetics in health care leads to a significant transformation in the organization of general health services, as it facilitates a shift from curative to preventive services [133]. The integration of genetics in general health services increasingly involves genetic aspects of clinical conditions, and as a result, the role of genetic and nongenetic professionals is changing [134]. More and more non-geneticist health care professionals are taking training and getting involved in genetic service provision [135]. However, as mentioned earlier, this practice is yet to be started in Bangladesh. In order to start genetic service in Bangladesh, the first step would be to initiate training for non-geneticist health professionals and infrastructure development.

Following the Human Genome Project's successful completion, healthcare technologies, especially the technologies related to medical genetic services, are developing faster than ever in the twenty-first century [136]. It is no surprise that the evolving technologies are shaping the practice of medicine, particularly the genetic service provision [137]. In order to start genetic service in Bangladesh, a dynamic and comprehensive guideline of genetic service provision would be needed. This guideline should encompass the challenges to integration, routine practices, personal and familial privacy of the service recipients, and regulation of technology application in the genetic service implementation [135, 137]. Also, the healthcare service infrastructure and operational facilities need to adapt or introduce new technologies [137]. Especially, operational facility competencies, including knowledge, attitude, and skills, are mandatory to achieve the transition efficiently [135]. This shift will require comprehensive training encompassing counseling and monitoring strategies besides the traditional focus on diagnostics and treatment in mainstream medicine. Active collaboration between scientists, healthcare professionals, and regulatory agencies needs to be established, and the recipients of the intended service should come forward with an inclusive and considerate mindset [138]. Otherwise, no such service provision system can be implemented [139]. Also, because genetic service is expensive in many cases, banking and insurance agencies need to develop health insurance policies that suit the socio-economic aspects of an LMIC like Bangladesh. Such health insurance policies may include sub-schemes for both mainstream and genetic medicine.

**Fig. 1:**
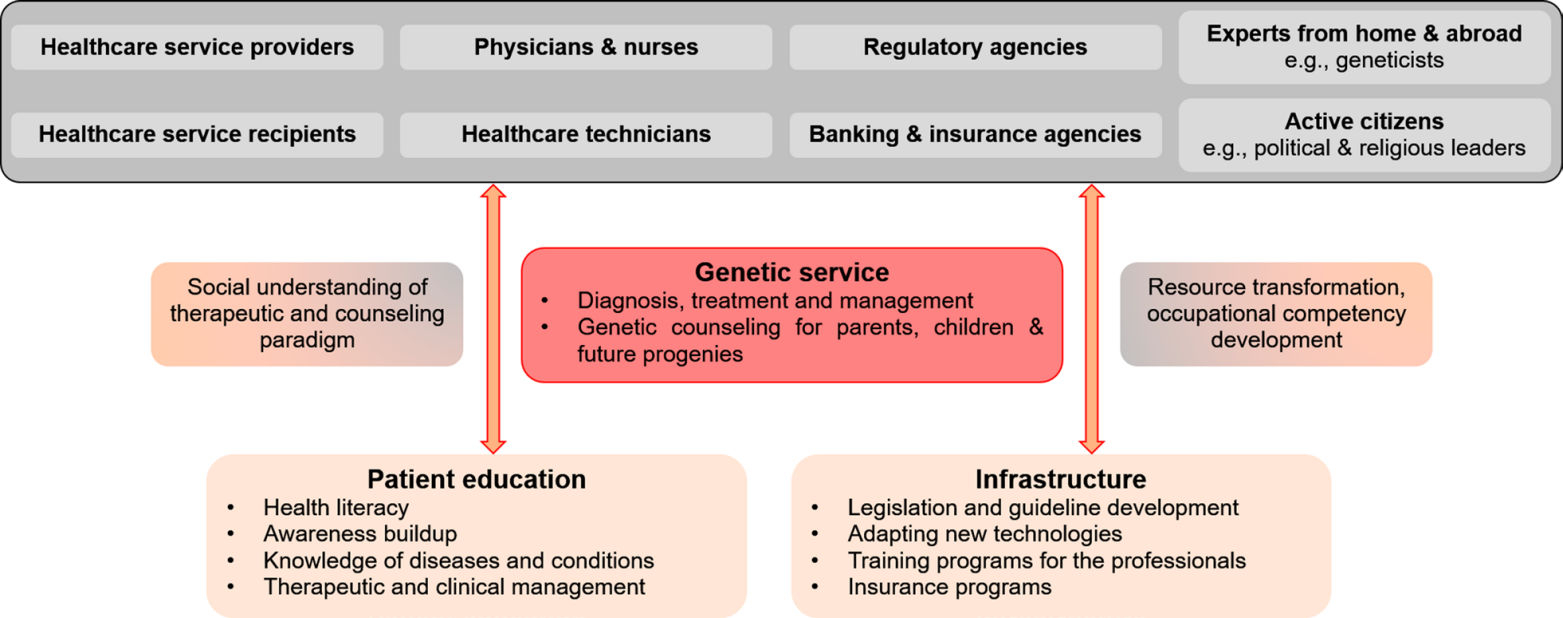
Patient education and infrastructure development on a genetic service implementation perspective in Bangladesh

A comprehensive nationwide survey to understand the epidemiology of genetic diseases, followed by patient registration and disease-wise database development, would be an essential assignment to start genetic service. Planning and implementing new patient education and awareness programs to educate people about the importance of genetic services, changing from a single-patient perspective to a family, converging the experience of the disease itself with the experience of being at risk for the disease is also an essential elementary step [140, 141]. The local experts who have hands-on training on medical and clinical genetics (academicians and researchers) can train the healthcare professionals in the country who do not have training in genetic medicine. The country may also call experienced educationists from foreign countries, who have expertise in genetic education and training implementation, to work with the local experts. Figure 1 shows a schematic of the perspective framework for genetic service implementation in Bangladesh.

Existing reports suggest that the high-throughput molecular diagnostics for thalassemias, which are rather costly in the context of LIC and LMICs, could be avoided in most cases if simpler and cost-effective methods could be utilized efficiently [51]. Assessing the hematological indices can be a good start for differential diagnosis of thalassemias [142]. However, a precise and accurate diagnosis of thalassemic conditions would require more sophisticated technologies, e.g., measuring the HbA2 concentration of lysed RBCs via HPLC, serum iron levels, and a ferritin calculation for ß-thalassemia [142, 143].

Since the development of the polymerase chain reaction (PCR) technique in the early 1970s, its use in pathological experiments is continually increasing [144]. Some variants and simple improvisations of PCR-based genotyping techniques could diagnose a significant majority of pathological mutations [144]. The development of specific primer panel based on comprehensive molecular epidemiological studies followed by multiplex, ARMS- and gap-PCR, and gel electrophoresis could diagnose most of the cases [145–148]. Results of pre-validated ARMS- and gap-PCR tests can be regarded as the gold standard for common thalassemic conditions; however, if the facility exists, Sanger or next-generation sequencing techniques can further strengthen the diagnosis.

Once facilities for hematological testing and/or primary molecular analysis become accessible to most of the community, the clinicians can then prescribe panel screening for specific diseases like thalassemias. To achieve this goal, we propose a collaborative model within the existing facility in Bangladesh, described in the latter part of this manuscript. Also, we considered the critical impact of individual-, family-, cultural-, institutional-, or policy-level factors that may influence patient outcomes of genetic counseling [149], including patient and genetic service provider's socio-cultural and educational backgrounds, disease-related elements, patient's health literacy level and clinical representation, family structure and history, economic status and type, social support, existing health policies, legalities and ethical aspects, health care infrastructure, and provider self-awareness.

In countries with limited and/or underutilized countries, like Bangladesh, the government needs to concentrate more on infrastructure and legal framework development. For example, the government can enforce laws for premarital screening for common recessive disorders, e.g., ß-thalassemic alleles, and advocate against consanguineous marriage. Indeed, the Bangladesh government has recently issued a rule for the premarital screening of hemoglobin variants [150]. However, its implementation is still not in place, and the diagnostic facilities are yet to be ready.

### A physician-academician collaborative model to start genetic counseling in the context of Bangladesh

**Fig. 2:**
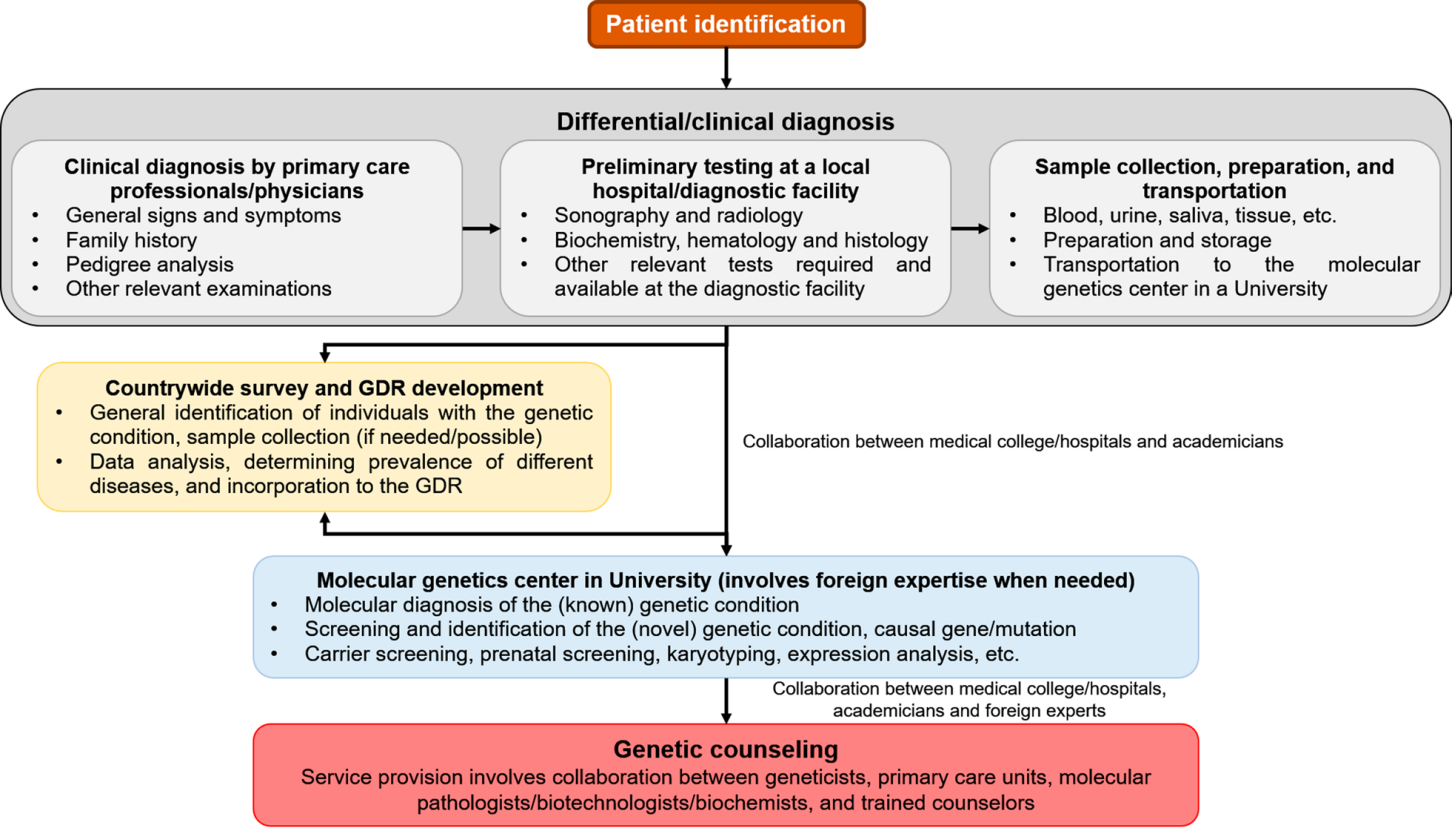
Model for implementing genetic diagnostics and counseling in Bangladesh. Patients will be identified, and histopathological and biochemical tests will be performed using the available facilities of the collaborating medical colleges. The molecular analysis will be performed at the genetic facilities of the university and foreign collaborators. This “Medical College-University-Foreign expert” model can be an effective model for setting up genetic counseling service provision systems in low-income and lower-middle-income countries. *GDR* genetic disease resource

Genetic diagnosis, testing, and counseling are real challenges in Bangladesh due to the factors mentioned above. Additionally, professionals, including the clinical geneticists, laboratory geneticists, bioinformaticians, genetics counselors, who work together in the completely established medical genetics facilities in HIC and UMICs, have not developed in the country. To tackle these challenges, we propose a framework to initiate a diagnostic and counseling service in Bangladesh (Fig. 2). We have currently established collaborations for our studies on the molecular screening of thalassemias, heritable arterial diseases, and diabetes in Bangladesh. We have also established such collaborations for studying some rare diseases in the country, including cystic fibrosis, myositis ossificans, and Hutchinson-Gilford progeria syndrome.

Our proposed framework is established on the concept that robust cooperation and collaboration, on both international and national levels, are key to success in implementing genetic services in the LICs and LMICs like Bangladesh. In this model, the primary care units, involving the primary care physicians (general practitioner or family physicians), nurse, physician associates, and support professionals, plays the most prominent role in genetic service provision. In these units, primary care units will be provided training in human genetics. In Bangladesh, non-government organizations and the government organize many workshops, seminars, and training programs for doctors, nurses, and health associates. The government itself and pharmaceutical companies in Bangladesh usually sponsor these workshops and training programs. The primary care units can receive basic training through multiple comprehensive training programs, instructed by medical genetics and biotechnology experts from home and abroad. Once they complete the basic clinical genetics training, the primary care physicians will undertake an initial risk assessment using a standardized referral guideline. The primary care physicians will identify the patients with potential genetic conditions or individuals at-risk using their expertise and available diagnostic facilities. They will then refer the patients with potential genetic conditions and individuals at-risk for genetic services by experts in medical genetics for further consultation. The geneticist will assess the patients’ condition and, if required, will suggest genetic testing at a designated laboratory, perhaps at a university or a research center, where the tests can be performed. In case the patient faces difficulty visiting the laboratory by himself, they can provide the sample to a local diagnostic or hospital facility (under the supervision of the primary care physician), where the sample will be processed, and they will send the sample to the designated lab. Next, the designated lab will perform genetic testing and inform the outcomes to the primary care unit and consulting geneticist. Based on the genetic testing outcomes, the patient (and family members) will receive a suggestion about the possible management strategies and treatment options. The suggestion they would be given will comply fully with the country's socio-cultural, ethical, and legal standards. For complicated cases, further testing and expert opinion could be sought from home and abroad. The pathway associated with this model is as follows: patient → primary care units → geneticist → laboratory → geneticist/primary care unit/professional counselor → patient and family members. A central coordination directory maintained by the authority of health services and society of medical genetics will regulate this pathway's effective management.

A major question that comes with this model is that who would pay for the genetic services? For the patients who have access to full coverage through social security or health insurance scheme, the service's cost could be covered by the scheme; however, it may require reviewing the coverage policies. A major challenge would be to finance the services for those who do not have access to such health coverage schemes. In such scenarios, the well-off households can finance the expenses from their savings or reduce on luxury stuffs of consumption [131, 151]. However, the less well-off households may need to cut back on daily necessities and reduce living budgets [151, 152]. Impulsive expenses for genetic services can expose households to a substantial financial risk, which is perhaps the most significant caveat of this model. The financial burden could be relieved by pointing and provisioning a public healthcare system or motivating the citizens to come under social security schemes [151]. Many previous studies have indicated that the policymakers must think effectively to develop and adapt systems to achieve national-level universal health coverage policies in Bangladesh [151, 153]. Given the great expense of genetic services, its implementation should be financed by the government through a national-level universal health coverage policy. However, the development and implementation of a national-level health policy for the citizens of Bangladesh depend on enhancing the budgetary allocation in the health sector and ensuring proper utilization of the allotted share. Long‐term sustainable investment and management of existing resources for effective implementation of genetic services in Bangladesh.

In the context of Bangladesh, medical doctors have direct access to handle patients, and most of the medical colleges/hospitals are equipped with different facilities, including radiology, biochemistry, hematology, histology, etc., for patient diagnosis. Nevertheless, within the existing situation, medical colleges/hospitals have insufficient facilities and lack of expert personnel for molecular diagnosis, especially for medical genetics work. On the other hand, many universities of Bangladesh have expert academics and molecular diagnosis facilities, but the academics depend on medical doctors for the subject cases. In this situation, if doctors at the medical colleges/hospitals make the primary or differential diagnosis with their facilities and collaborate with nearby university academics for molecular diagnosis, it can be a collaborative tire model for proper diagnosis. If medical doctors from all over the country, molecular biologists, geneticists, and bioinformaticians with training in genetic diseases can make a collaborative group, they can start genetic counseling programs. Most of the academics and many medical doctors also pursue their higher studies abroad with reputed medical colleges or universities, and most of them have a good network with international clinicians and expert groups. If necessary, for any complicated case of genetic disease diagnosis, treatment, or management, a foreign expert group's opinion can also be asked. In these collaborative ways, within the existing facilities, it is possible to mitigate many issues related to genetic diseases.

One of the challenges in this model would be to distinguish between genetic and non-genetic conditions that are coexisting in the country. The general physicians, who will primarily report identify a possible genetic condition, would be relying on the routine clinical/non-genetic diagnostic techniques, e.g., complete blood count, liver function tests, kidney function tests, etc. Complete resources, like the GeneReviews®, on the clinical representations of all the genetic diseases available in the country, are required to be developed [154]. However, this kind of resource for geographic area-specific manifestations is not yet available in Bangladesh and other LICs and LMICs. Our group is currently working to note the general clinical manifestations of frequent genetic diseases in Bangladesh. As we have already worked on a couple of nation-wide field surveys, we have developed some good communication to the urban and rural areas of Bangladesh. These communications will help obtain epidemiological data and the prevalence of genetic diseases in the country. Besides, some other groups are also working in this area in the country. More comprehensive activities by other academicians, both individually and collaboratively, would fasten the process and enhance our knowledge of the country's clinical representations of different genetic diseases. When clinical data on most genetic disorders are reported, a compilation of the reports could be used as a comprehensive genetic disease resource (GDR). For efficient maintenance and to ensure continuing the inclusion of relevant and medically important contents into the GDR, a dedicated team affiliated with the Bangladesh Bureau of Educational Information and Statistics will be given charge.

When this resource will be ready for clinical use, the clinicians will have free access to this resource. Subjects representing clinical conditions resembling any of the listings in the GDR and meeting any of the following criteria should be contemplated for a referral to genetic diagnosis:

- The subject has a positive family history
- The subject belongs to a consanguineous family
- The subject has developmental delays, which is, perhaps, due to an inherited or congenital issue
- The subject him/herself or his/her parents have a reproductive complication

Until the geographic location-specific GDR comes prepared for clinical use, the clinicians can still start this type of program with having reference to the GeneReviews®. For novel and extremely rare genetic conditions, subject samples could be analyzed using an established gene-discovery strategy by an interested academic (Table 2) [6].

**Table 2:** Strategies for gene discovery

Characteristics	Approach to analysis
Unrelated individuals or families affected by the same very rare but highly relatable clinical condition	Identify a common disease-associated gene or pathway shared between unrelated affected individuals
Inbred (e.g., consanguineous) families	Map based on homozygosity to exclude most of the genome
Autosomal-dominant families	Map to exclude most of the genome
Non-inbred families with two or more affected siblings	Identify compound-heterozygous variants (in the same gene) shared between affected siblings
Single affected individuals with no family history	Identify deleterious variants in genes with disease associations

Finally, genetic disease awareness and its pre-conceptional implications should be disseminated through leaflets, audio–video advertisements, TV programs, and by introducing it in the secondary level curriculum. Social activists, social workers, and religious and political leaders will also be provided with sufficient knowledge and asked to help disseminate scientifically factual information on genetic diseases among mass populations. For instance, the Imams (a Muslim leadership position) of each mosque could play a vital role in disseminating awareness about genetic conditions. Besides, in the last decade, Bangladesh has established more than 13,000 community clinics to provide primary healthcare in rural areas [155]. These community clinics will play a vital role in educating the people in rural areas. In collaboration with other government and non-government bodies, the Bureau of Health Education will coordinate the patient education and awareness programs. Advocacy initiatives targeting the social security officials and private insurance companies also need to be propagated. In the long run, for sustainable implementation of genetic services, training programs conferring institutional degrees, e.g., diploma, master's degree, are required to be started by the authorities.

## Conclusions

The incidence of genetic diseases in Bangladesh is exceedingly high, in part due to the high rate of consanguinity and chronic exposure to various environmental pollutants. Knowledge among the general population about premarital screening and genetic counseling programs is low and needs to be promoted, with educational campaigns in hospitals, schools, and universities. The provision of genetic screening with advanced technology and counseling in government-regulated facilities for all couples wishing to marry remains essential. To be effective, counseling should be provided by trained professionals who can provide accurate advice regarding the risks and childbearing options. This will require an additional focus on genetics and genetic disease in medical education but will also require implementation-specific courses for genetic counselors. Though time demanding, the start of a genetic counseling service at least in each of the district hospitals will help to better management of disease and will decrease the prevalence of genetic diseases in Bangladesh. If this framework proves efficient, the framework on which it is based can be used as a starting point for other LICs and LMICs to develop genetic services.

## Data Availability

All data collected and produced in this study that supports the conclusions of this article is included within the article.
